# The Role of Rac1 in the Growth Cone Dynamics and Force Generation of DRG Neurons

**DOI:** 10.1371/journal.pone.0146842

**Published:** 2016-01-14

**Authors:** Wasim A. Sayyad, Paolo Fabris, Vincent Torre

**Affiliations:** Neuroscience Area, International School for Advanced Studies (SISSA), Trieste, Italy; Rutgers University, UNITED STATES

## Abstract

We used optical tweezers, video imaging, immunocytochemistry and a variety of inhibitors to analyze the role of Rac1 in the motility and force generation of lamellipodia and filopodia from developing growth cones of isolated Dorsal Root Ganglia neurons. When the activity of Rac1 was inhibited by the drug EHop-016, the period of lamellipodia protrusion/retraction cycles increased and the lamellipodia retrograde flow rate decreased; moreover, the axial force exerted by lamellipodia was reduced dramatically. Inhibition of Arp2/3 by a moderate amount of the drug CK-548 caused a transient retraction of lamellipodia followed by a complete recovery of their usual motility. This recovery was abolished by the concomitant inhibition of Rac1. The filopodia length increased upon inhibition of both Rac1 and Arp2/3, but the speed of filopodia protrusion increased when Rac1 was inhibited and decreased instead when Arp2/3 was inhibited. These results suggest that Rac1 acts as a switch that activates upon inhibition of Arp2/3. Rac1 also controls the filopodia dynamics necessary to explore the environment.

## Introduction

Neurons are specialized cells responsible for exchanging information with other neurons or cells through synapses [1]. During development, differentiating neurons explore the surrounding environment in order to form the correct contacts and they use highly motile structures called growth cones (GCs) located at the tip of their neurites [2,3]. GCs consist of a flat extension, named ‘lamellipodium’ with varying width from which finger-like submicron diameter structures called filopodia emerge [4]. The process of polymerization of actin filaments is the main source of GC protrusion, which is regulated and controlled by several proteins such as Arp2/3, cofilin, formin and molecular motors, such as myosin, dynein, controlling different features of cellular motility [5].

Actin related protein 2/3 complex (Arp2/3) is widely studied for its involvement in lamellipodia formation and protrusion [6,7]. Arp2/3 consists of seven subunits and promotes the formation of branched actin filament networks [8,9]. Arp2/3 not only regulates the branching of actin filaments but it is also involved in the formation and dynamics of filopodia [10,11]. Inhibition of Arp2/3 causes lamellipodia retraction and an increase of the actin retrograde flow rate [10]. Arp2/3 is inactive in its native state and the members of the Wiskott-Aldrich syndrome protein (WASP) family, downstream of Rac and Cdc42 pathways activate the Arp2/3 complex to nucleate new filaments [12,13]. Rac binds the WAVE (WASP family Verprolin Homology Domain-containing protein) complex to release active WAVE, which promotes actin polymerization through activation of Arp2/3. WASP and WIP (WASP-interacting protein), downstream effectors of Cdc42 interact directly with Arp 2/3 complex to promote filopodia formation. Recently a new protein called Arpin has been shown to be part of the Rac-Arpin-Arp2/3 inhibitory circuit playing a major role in steering during cell migration [14]. Rac can both activate and inhibit Arp2/3-driven actin branching and polymerization to regulate speed, directionality and persistence of membrane protrusions.

Rho family GTPase has distinct and specific roles in the regulation of growth, maintenance and retraction of GCs [15]. The mammalian Rho GTPase family currently consists of three subfamilies, Rho (RhoA, RhoB and RhoC), Rac (Rac1, Rac2 and Rac3) and Cdc42 (Cell Division Cycle-42) (Cdc42Hs and G25K). RhoA, Rac1 and Cdc42 are well-studied members of Rho family GTPase controlling distinct cytoskeletal elements. Activation of Rac1 stimulates actin polymerization to form lamellipodia, Cdc42 induces the polymerization of actin to form filopodia or microspikes which are parallel actin bundles within the lamellipodium and Rho regulates the bundling of actin filaments into stress fibers and the formation of focal adhesion complexes. The Rho family of GTP-binding proteins are activated by a variety of growth factors, cytokines, adhesion molecules, hormones, integrins, G-proteins and other biologically active substances [15,16]. Biochemical approaches or analyses of the morphology of fixed cells have shown that Rho GTPase also involves crosstalk. This may occur through the Rac/Cdc42 effecter PAK, which can negatively regulate Rho GEFs [17] or other mechanisms including, via reactive oxygen species [18], phosphorylation and competitive binding of RhoGDI [19] or binding of GEFs to actomyosin[20]. Depending upon the concentration and localization of these Rho GTPase, mammalian cells show different morphology, movement and behavior [21].

When the rate of actin polymerization overtakes the actin retrograde flow, the GC protrudes [22]. Retrograde flow refers to the backward flow of the actin filament network away from the growth cone leading edge into the C-domain. This allows the addition of actin monomers/oligomers to actin filaments in close contact with the membrane, pushing the cellular membrane forward, leading to the protrusion. Mitchison and Kirschner proposed the ‘Molecular Clutch Hypothesis’, which postulates that an intracellular molecular clutch, formed by interactions between GC membrane adhesive receptors and the extracellular environment, couple to the overlying flow of actin filaments to slow down their retrograde rate[23]. Formation of these ‘clutches’ together with myosin II contractile activity, provides a traction to pull and move the central region of the GC closer to the peripheral region, leading to axonal lengthening. Therefore, substrate adhesion decreases the actin retrograde flow. The decrease in the actin retrograde flow together with actin polymerization, results in the leading edge protrusion[24,25].

The traction force is essential for cell migration, cell shape maintenance, mechanical signal generation and other cellular functions. There are different methods to quantify the cellular traction forces. Traction force microscopy measures the stress of a cell on an elastic gel substrate by detecting the movement of fluorescent beads embedded at the surface of the gel [24]. With Optical Tweezers the bead is attached to the cell membrane either to apply the tensile force [25,26] or to measure the retrograde flow rate [27]. We have independently developed a method to estimate the force exerted by the lamellipodia and filopodia by measuring the displacement of the bead using quadrant photo detector (QPD) [28–33]. In our case, the bead is not initially attached to the cell membrane and it is kept in the vicinity of the lamellipodia or filopodia, so that their spontaneous motion can displace the bead.

We observed that the lamellipodia transiently retract and recover back after 5–8 min of Arp2/3 inhibition. In this study we have investigated the role of Rac1, in the recovery of lamellipodia in Arp2/3 depleted condition and also in GC motility, by using Optical Tweezers and specific inhibitors of Arp2/3 (CK-548) and Rac1 (EHop-016 and F56). Motility of lamellipodia and of filopodia was also followed and characterized by video imaging. By combining these techniques together with immunofluorescence we have explored the interaction between Rac1 and Arp2/3 complex and their role in the formation of lamellipodia and filopodia of Dorsal root Ganglion (DRG) GCs. Here we show that Rac1 acts as a switch and activates upon inhibition of Arp2/3.

## Results

After 6–8 hours of culture, differentiating DRG neurons have neurites emerging from their soma. At the tip of the protruding neurites, GCs lamellipodia and filopodia explore the environment and their motion continues for 1–3 days. The motility of lamellipodia and filopodia slows down when appropriate connections are established and the neuronal network is formed; the leading edge of these lamellipodia can move with a speed 30–100 nm/s exerting a force exceeding 20 pN [32]. The effect of the inhibitors of specific proteins involved in the regulation of GC motility was analyzed after 24–48 hours of culture, when the motility of filopodia and lamellipodia was more pronounced. We focused on the analysis of inhibitors of small GTPases and of the Arp2/3 complex.

**Fig 1:**
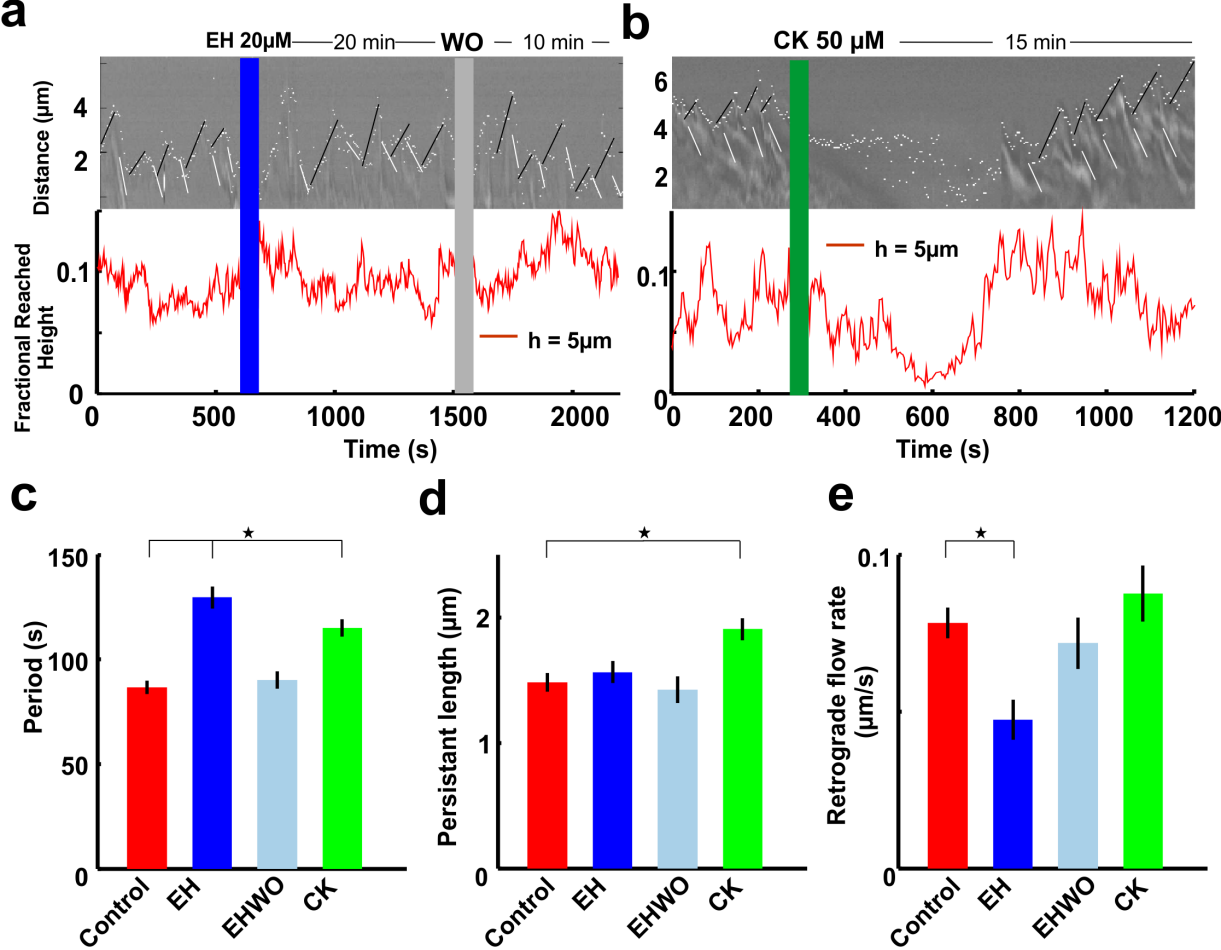
The effect of Rac 1 and Arp2/3 Inhibitor on the motility of lamellipodia. **(a)** Kymograph (upper panel) showing the protrusion/retraction cycles of lamellipodia in control conditions (before the blue vertical line), 20 μM EH (after the blue vertical line), followed by washout (after the grey vertical line). Fractional height (lower panel) reached by lamellipodia before and after 20 μM EH, followed by washout. **(b)** Same as in (a) but in the presence of 50μM CK (after the green vertical line) without washout. White dots show the leading edge of lamellipodia. Descending white lines label retrograde flow of lamellipodia and ascending black lines indicate lamellipodia protrusion. **(c)** Period of protrusion/Retraction cycles of lamellipodia in control conditions, with 20 μM EH (20 min), after washout of 20 μM EH (10 min) and 50μM CK (15 min, after recovery). **(d)** Persistence length of lamellipodia in control conditions, with 20 μM EH (20 min), after washout of 20 μM EH (10 min) and 50μM CK (15 min, after recovery). **(e)** Retrograde flow rate of lamellipodia in control conditions, with 20 μM EH (20 min), after washout of 20 μM EH (10 min) and 50μM CK (15 min, after recovery). Student t-test showed that the data significantly differ from the control conditions, *P<0.05. Data represent mean ± SEM.

We used the small molecules CK-636, CK-548, CK-666 and CK-869 as inhibitors of the Arp2/3 complex. All these compounds at a high concentration, i.e. above 100 μM, abolished GC motility completely and in the experiments here described we used extensively CK548 (CK) as the Arp2/3 inhibitor, since CK decreases the affinity of rhodamine-N-WASP-VCA for BtArp2/3 complex approximately twofold [34]. Furthermore, we tested two inhibitors of Rac1 namely, EHop-016 (EH) [35] and F56 [36] and the Cdc42 inhibitor ZCL-278 (ZCL) [37]. In addition to these drugs, CT04 (CT) [38] and GSK 269962 (GSK) [39] were also used as inhibitors of the RhoA and Rock pathways respectively. In order to check if the effect of the inhibitors was a side effect of toxicity we also checked their reversibility after washout (WO), as shown in Fig 1.

### The effect of partial inhibition of Rac1 and Arp2/3 in lamellipodia motility

The involvement of Rac1 and Arp2/3 in lamellipodia motility of DRG GCs was studied by analyzing the effect of their inhibitors EHop-016 (EH) and CK-548 (CK) respectively and by quantifying lamellipodia motility using the two algorithms as described in the Materials and Methods section, based on the analysis of Z-stack phase contrast video imaging. From the image sequences, kymographs were obtained by using algorithm I. The ability of lamellipodia to lift up vertically was quantified by computing the fraction of pixels in focus at 5 μm above the coverslip obtained by using algorithm II (Fig 1A and 1B).

When Rac1 activity was inhibited by 20 μM EH lamellipodia still exhibited protrusion retraction cycles (Fig 1A, upper panel) and could lift up in the axial direction (Fig 1A, lower panel). EH effects were reversible and period, persistence length, retrograde flow rate of lamellipodia returned to control level after washout (Fig 1C–1E). Interestingly, lamellipodia of DRG GCs, treated with 50 μM CK showed a transient retraction and were not able to lift up vertically in a significant manner. However, treated lamellipodia recovered their usual motility in 5–8 min (Fig 1B, upper panel) and were able to lift up in the axial direction as in control conditions (Fig 1B, lower panel). Following 50 μM CK treatment, period, persistence length and retrograde flow rate of lamellipodia were quantified during lamellipodia recovery period. The average period of protrusion/retraction cycles of lamellipodia increased significantly, both in the presence of 20 μM EH (129.6±5.2 s) and of 50 μM CK (115.1±4.2 s) respectively compared to control conditions (86.5±3.2 s) and after washout of 20 μM EH (90.3±4.1 s) (Fig 1C). The persistence length of lamellipodia i.e the maximum extension reached by the lamellipodia after which they start to retract, increased when Arp 2/3 was inhibited by 50 μM CK (1.90±0.09 μm) compared to control conditions (1.48±0.07 μm) and after washout of 20 μM EH (1.42±0.1) (Fig 1D). However, there was no significant change in the persistence length of lamellipodia when Rac1 was inhibited (1.56±0.09 μm), but the lamellipodia retrograde flow rate decreased when Rac1 was inhibited (0.05±0.007 μm/s) compared to what observed in control conditions (0.08±0.005 μm/s), after washout of 20 μM EH (0.07±0.008 μm/s) and in the presence of Arp2/3 inhibitors (0.09±0.009 μm/s) (Fig 1E).

### Rac1 activates when Arp2/3 is inhibited

**Fig 2:**
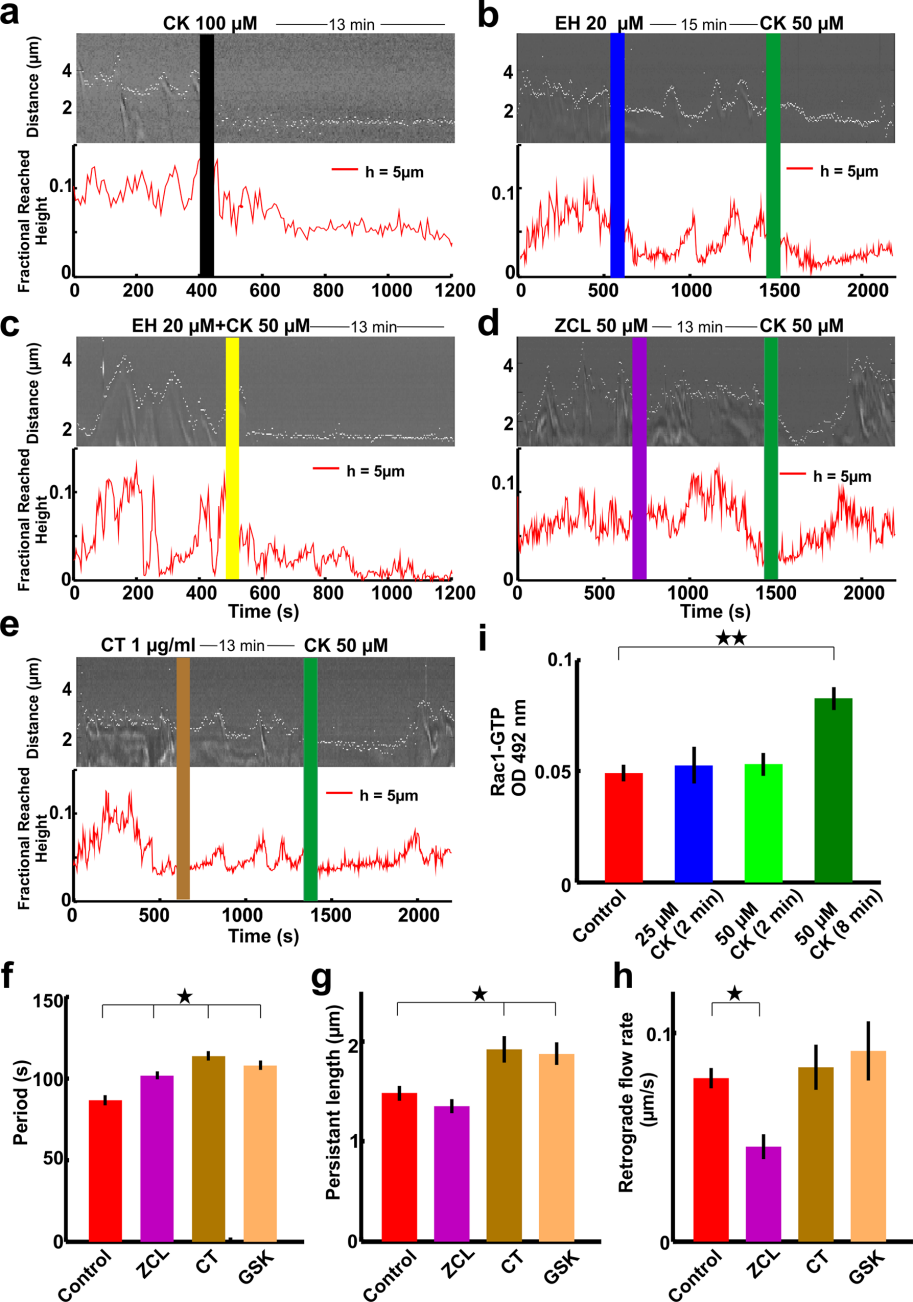
Rac1 restores lamellipodia’s motion after transient retraction when Arp2/3 is inhibited. **(a)** Kymograph (upper panel) and fractional height reached by lamellipodia (lower panel) in control conditions (before the black vertical line) and in the presence of 100 μM CK (after the vertical black line). **(b)** As in (a) but in the presence of 50 μM CK (green line) and of 20 μM EH (blue line). **(c)** As in (a) but in the presence of 50 μM CK and of 20 μM EH together (yellow vertical line). **(d)** As in (a) but in the presence of 50 μM ZCL (purple vertical line) and of 50 μM CK (green vertical line). **(e)** As in (a) but in the presence of 1μg/ml CT04 (brown vertical line) and 50 μM CK (green vertical line). Vertical lines show time at which the inhibitors were added. We observed the same behavior in each case for n ≥ 8 experiments. **(f)** Period of protrusion/Retraction cycles of lamellipodia in control conditions, with 50 μM ZCL (13 min), with 1μg/ml CT (13 min) and 500nm GSK (13 min). **(g)** Persistence length of lamellipodia in control conditions, with 50 μM ZCL (13 min), with 1μg/ml CT (13 min) and 500nm GSK (13 min). **(h)** Retrograde flow rate of lamellipodia in control conditions, with 50 μM ZCL (13 min), with 1μg/ml CT (13 min) and 500nm GSK (13 min). Student t-test showed that the data significantly differ from the control conditions, *P<0.05. Data represent mean ± SEM. **(i)** Quantification of Rac1-GTP level in DRG neurons in control, 25 μM CK (after 2 min), 50 μM CK (after 2 min) and 50 μM CK (after 8 min) conditions. Student t-test showed that the data significantly differ from the control conditions, n = 8,**P<0.005. Data represent mean ± SEM.

When the activity of Arp2/3 was inhibited by 100 μM of CK lamellipodia shrank and their motility was completely and permanently suppressed (Fig 2A). The growth cone also lost the adhesion to the substrate and retracted towards the soma (data not shown). Remarkably, when DRG neurons were treated with 50 μM CK, lamellipodia showed a transient retraction that continued for 5–8 minutes, but then lamellipodia recovered their usual motility restoring protrusion and retraction cycles and were also able to lift up vertically almost as under control conditions (Fig 1B). The results of these experiments suggest that following a partial inhibition of Arp2/3 another pathway is activated rescuing—to some extent—the usual GC motility. To test this possibility and to identify the origin of the recovery of motility in treated lamellipodia, we considered the Rho GTPase pathways, known to regulate many aspects of intracellular actin dynamics and GC metabolism [40]. The most extensively studied members of Rho GTPase family are Rho A, Rac1 and Cdc42. Rac can not only regulate actin polymerization but it can also increase the availability of free actin-barbed ends by the removal of capping proteins and it can also increase the availability of actin monomers by regulating cofilin [41]. These roles of Rac1 could help in the formation and protrusion of lamellipodia by polymerizing the pre-existing branched actin filaments at the leading edge of the lamellipodia, in Arp2/3 depleted condition. In addition, the newly formed actin branches generated by the remaining Arp2/3 can contribute to the lamellipodia protrusion. Therefore, we hypothesized that Rac1 could mediate the recovery of motility observed in Fig 1B. Lamellipodia that were first treated with 20 μM EH exhibited an increase in the period of protrusion/retraction cycles and could move up in the axial direction (Fig 1A and 1C). Then, the same lamellipodia were treated also with 50 μM CK: in this case, as expected, lamellipodia shrank but could not recover their motility even after 10–20 minutes of exposure to these inhibitors (Fig 2B). We tested also the simultaneous application of 20 μM EH and of 50 μM CK, which were mixed and added to the medium bathing of the neuronal culture at the same time. Lamellipodia exposed simultaneously to the two inhibitors retracted and did not show any sign of motility even after 10–20 minutes (Fig 2C).

The above results indicate that Rac1 could be behind the recovery of lamellipodia which were transiently retracted after Arp2/3 inhibition. However, the Rac1 inhibitor EHop-016 inhibits both Rac3 as well as Cdc42 above the concentration of 3μM[35]. Therefore, in order to examine the possible role of the Cdc42 pathway, we used ZCL as a selective inhibitor which is known to target the binding site of the Cdc42 guanine nucleotide exchange factor, intersectin (ITSN) and to hinder Cdc42 activation[37]. When 50μM ZCL was added lamellipodia did not show significant changes in their motility. Subsequent exposure of 50 μM CK to the same lamellipodia shrank the lamellipodia as usual, but then lamellipodia did recover after approximately 8 minutes of exposure (Fig 2D).

To dispose the possibility of Rac3, in the recovery of lamellipodia, in Arp2/3 inhibited condition, we used F56 as another specific Rac1 inhibitor. It is a control peptide version of Rac1 Inhibitor W56; comprises residues 45–60 of Rac1 with Trp56 replaced by Phe, which does not affect GEF-Rac1 interaction[36]. When lamellipodia were treated with 100μM F56, lamellipodia did not show significant changes in their motility. The same lamellipodia were then exposed to 50 μM CK, the lamellipodia shrank as usual, but then lamellipodia did not recover even after 10–20 minutes of exposure. We tested also the simultaneous application of 100 μM F56 and of 50 μM CK, which were mixed and added to the medium bathing of the neuronal culture at the same time. Lamellipodia exposed simultaneously to the two inhibitors retracted and did not show any sign of motility after 10–20 minutes (data not shown).

In the Arp2/3 depleted situation, in order to see the role of RhoA and Rock in the lamellipodia recovery, lamellipodia were exposed to CT (Rho A inhibitor)[38] (Fig 2E) and GSK (ROCK inhibitor) [39] (data not shown) independently, before the treatment with CK. In both situations lamellipodia recovered after 8 minutes of exposure and, at the end of their retraction, they were also able to reach the same height as in control conditions. We analyzed in detail the growth cone dynamics in the presence of the inhibitors of CDC42, RhoA and ROCK signaling pathways. The period of lamellipodia protrusion/retraction cycles in the presence of ZCL (101.7±2.6 s), CT (113.6±2.9 s) and GSK (107.8±2.9 s) increased when compared with the control conditions (86.5±3.2 s) (Fig 2F). The lamellipodia persistence length also increased in the presence of CT (1.92±0.13 μm) and GSK (1.8±0.11 μm) but remained constant in the presence of ZCL (1.35±0.07 μm) when compared to control conditions (1.48±0.07 μm) (Fig 2G). The retrograde flow rate decreased in the presence of ZCL (0.05±0.006 μm/s) but remained constant in CT (0.08±0.01 μm/s) and GSK (0.09±0.01 μm/s) when compared to control conditions (0.08±0.005 μm/s) (Fig 2H).

These results discard the involvement of the Rac3, Cdc42, RhoA and Rock pathways in the recovery of lamellipodia which were transiently retracted after the inhibition of Arp2/3. It also indicates that Rac1 is crucial for the recovery of the transient retraction of lamellipodia due to inhibition of Arp2/3.

To confirm the role of Rac1 during the recovery of the transient lamellipodia retraction due to 50 μM CK, we examined the Rac1-GTP level (the activated form of Rac1) in cultured DRG neurons with different exposure conditions of CK (Fig 2I) by using the G-LISA Rac 1 activation assay kit (Cytoskeleton, Inc., Denver, Colo.) (see Materials and Methods). The Rac1-GTP level, in the presence of 25 and 50 μM CK for 2 minutes did not show any significant change compared to the control conditions. However, the Rac1-GTP level significantly increased in the presence of 50 μM CK for 8 minutes (P<0.005) i.e. the time during which lamellipodia recovered motility after the transient retraction (Fig 2I). These results confirm that Rac1 activates upon inhibition of Arp2/3.

### Effect of Arp2/3 and Rac1 inhibitors on the force exerted by lamellipodia

**Fig 3:**
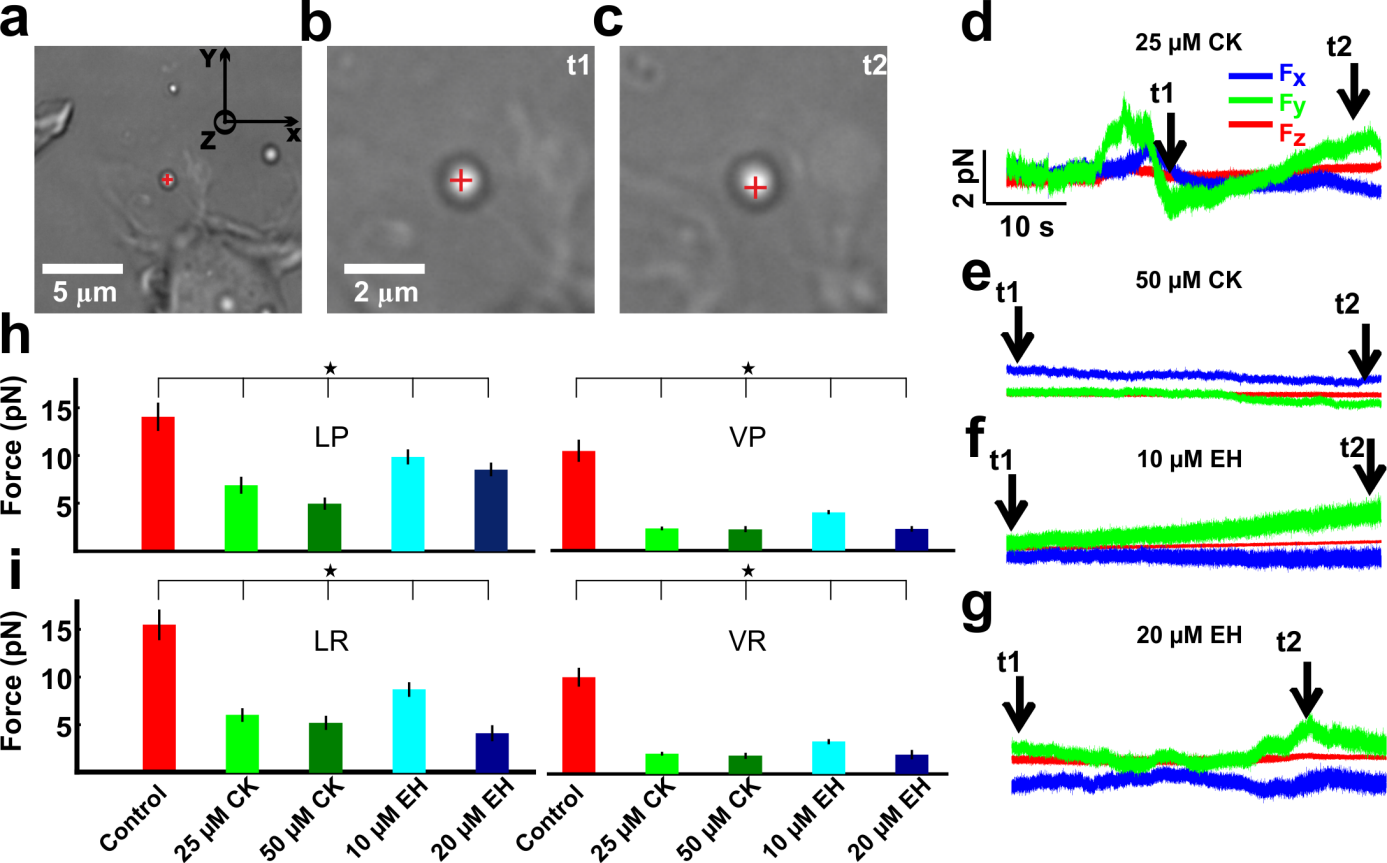
The effect of CK and EH on the force generated by lamellipodia. **(a)** Low-resolution image of a bead trapped in front of a lamellipodium emerging from the soma of a DRG neuron in the presence of 25 μM CK (25 μM CK). Scale bar, 5μm. (**b-c**) High-resolution images during a push. At t_1_ the bead is in the optical trap (b) and when the lamellipodium grows, at t_2_, it pushes the bead (c). The red cross indicates the centre of the optical trap. Scale bar, 2μm. (**d**) The three components F_x_, F_y_, and F_z_ of the force exerted when the lamellipodium pushes the bead. (**e**) As in (d) but in the presence of 50 μM CK (CK 50 μM). (**f**) As in (d) but in the presence of 10 μM EH (EH 10 μM). (**g**) As in (d) but in the presence of 20 μM EH (EH 20 μM). The trap stiffness is k_x,y_ = 0.10, k_z_ = 0.08 pN/nm. **(h)** Comparison of the force exerted by lamellipodia in control conditions (red), with 25 μM CK (green), with 50 μM CK (dark green), with 10 μM EH (cyan) and with 20 μM EH (blue) and in all the four different stereotyped behaviours: LP, LR, VP and VR. In each case, by using the student t-test, the force measured in the presence of each inhibitor was lower than that measured in control conditions with a significance *P<0.005. Data represent mean ± SEM.

Optical Tweezers was used to investigate the effect of the partial inhibition of Rac1 and Arp2/3 on the force exerted by lamellipodia. Lamellipodia in control conditions pushed the trapped beads with a force up to 10–20 pN as previously described [31] and often beads could be displaced out of the optical trap. The forces were measured from the same lamellipodia in control conditions and in the presence of the inhibitors. Exerted forces were analyzed according to four different stereotyped behaviors previously described [32,33], depending on the direction in which the lamellipodia were exerting the force on the bead: vertical push (VP), vertical retraction (VR), lateral push (LP) and lateral retraction (LR) (Fig 3).

Lamellipodia of DRG treated with a moderate concentration of Rac1 and Arp2/3 inhibitors were able to pull and push a trapped bead, but with a lower force compared to the force observed in control conditions (Table 1). In the lateral direction: in case of LP the lamellipodia force decreased by 30–40% with an increase in the inhibition of Rac1, however it decreased by 50–65% when Arp2/3 was inhibited compared to control conditions. The retractile force LR decreased by 40% when Rac1 was inhibited by 10μM EH, inhibition of Rac1 by 20 μM EH decreased the LR force more than 70% probably due to a decrease in the lamellipodia retrograde flow rate. The retractile force LR decreased by 65% when Arp2/3 was inhibited. In the axial direction: when Rac1 was inhibited by 10 μM EH, the lamellipodia force in VP and VR decreased more than 60%. Besides, it decreased more than 75% in all the other VP and VR cases (Table 1).

**Table 1:** The effect of different inhibitors on the force exerted by lamellipodia.

Force (pN) N ≥ 15	Control	EH 10 μM	EH 20 μM	CK 25 μM	CK 50 μM
**LP**	14.0±1.5	9.9±0.8	8.5±0.7	6.9±0.9	5.0±0.6
**VP**	10.4±1.2	4.0±0.2	2.3±0.3	2.3±0.2	2.2±0.3
**LR**	15.5±1.6	8.7±0.8	4.1±0.8	6.0±0.7	5.2±0.7
**VR**	10.1±1.0	3.4±0.3	2.0±0.5	2.1±0.2	1.9±0.3

Average maximum force exerted by lamellipodia in control conditions (second column), in the presence of 10 μM EH (third column), of 20 μM EH (fourth column), of 25 μM CK (fifth column) and of 50 μM CK (sixth column) for lateral push (second row), vertical push (third row), lateral retraction (fourth row) and vertical retraction (fifth row) respectively. The student t-test has shown that in all the cases, the data significantly differ with respect to control conditions, P<0.05. Data represent mean ± SEM.

These results suggest that lamellipodia were not able to explore the surrounding environment with an equal force when Rac1 and Arp2/3 were inhibited when compared to control conditions. In addition, lamellipodia were not able to exert a larger force in the axial direction than in the lateral direction, when compared with the control conditions state.

### The effect of Rac1 inhibitors on the rate of lamellipodia protrusion

Lamellipodia in the presence of 10–20 μM EH exerted a lower force but were still able to extend. In order to measure their rate of protrusion, we used the Nanopositioner feedback (see Materials and Methods section) which allows a precise and continuous measurement of the bead position by employing Optical Tweezers.

**Fig 4:**
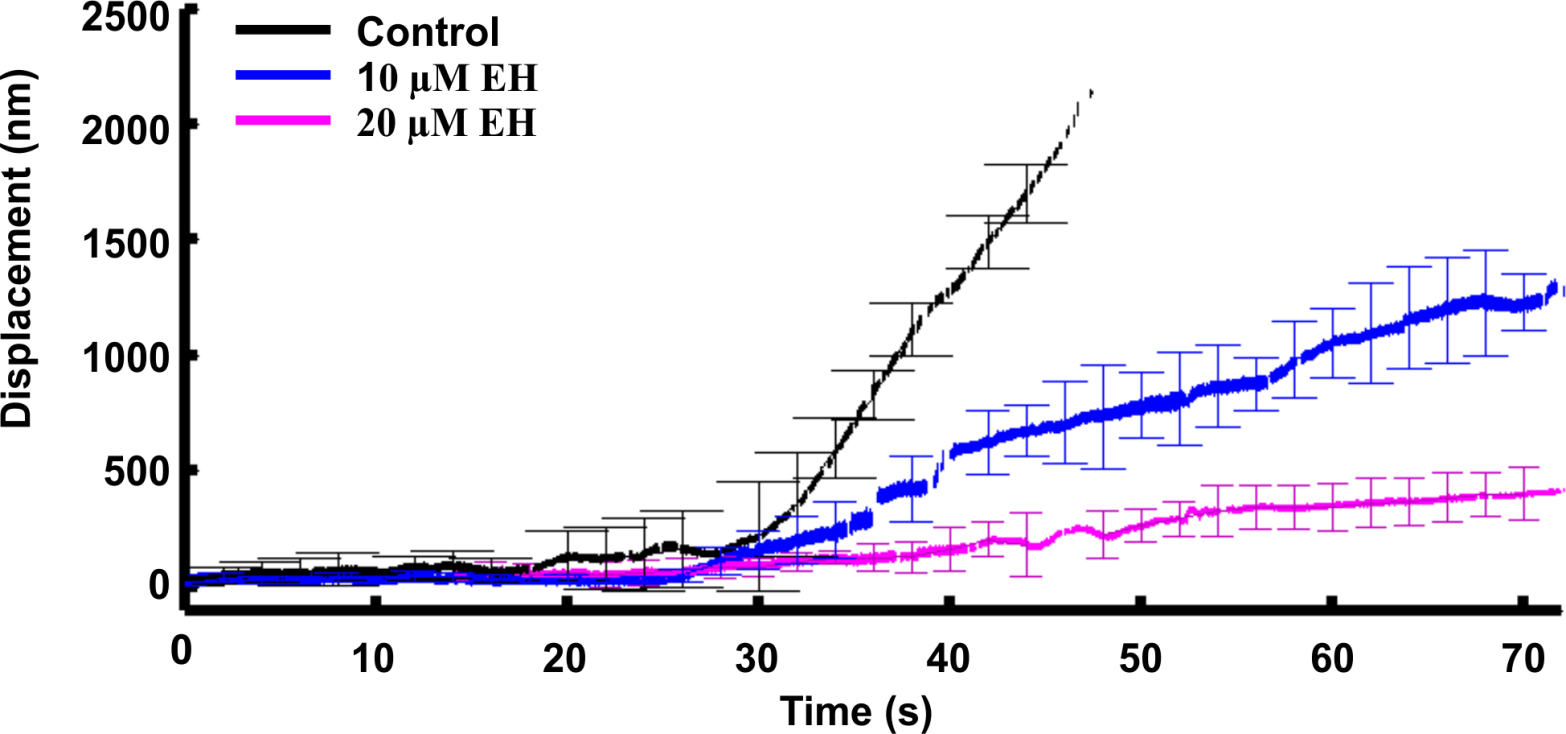
The rate of lamellipodia protrusion. To track the protrusion of lamellipodia, the position of the bead, displaced by the protruding lamellipodia, was followed using the feedback mechanism of the nanopositioner (See Results and Materials and Methods sections). The figure shows the total displacement of the bead in control conditions (black), with 10μM EH (blue) and with 20μM EH (magenta).

All the measurements obtained using the nanopositioner feedback mechanism were compensated as explained in the Materials and Methods section. In each case, the total displacement of the bead in the lateral direction was computed. In each case—control conditions, 10 μM EH and 20 μM EH- 5–6 of such traces were averaged and plotted against time (Fig 4). Before averaging, traces were aligned so that their rising phase matched each other. The slopes of these traces were calculated to determine the lamellipodia protrusion rate. In control conditions, the speed of protrusion of lamellipodia could reach 100 nm/s (see black trace in Fig 4) and was reduced to 30–50 nm/s in the presence of 10 μM EH (blue trace in Fig 4) and to 10–20 nm/s in the presence of 20 μM EH (magenta trace in Fig 4).

These results indicate that inhibition of Rac1 has a similar effect on the amplitude of the force exerted by lamellipodia and on their protrusion rate.

### Effect of Arp2/3 and Rac1 inhibitors on the force exerted by filopodia and their motility

The filopodia motility and the force exerted by them were quantified by video imaging, immunocytochemistry and Optical Tweezers (Table 2). The protruding filopodia tips were followed in different frames to calculate the filopodia protrusion rate, and the maximum length of the filopodia was measured as described in the Materials and Methods section.

**Table 2:** Filopodia motility and force exerted by them.

Filopodia	Control	CK 25 μM	CK 50 μM	EH 10 μM	EH 20 μM
**Length (μm)**	3.36±0.2	5.67±0.25*	6.33±0.3**	4.14±0.18**	8.04±0.39**
**Growth rate (μm/s)**	0.10±0.001	0.09±0.004	0.07±0.003**	0.09±0.005	0.13±0.004**
**Force (pN)**	3.08±0.15	2.74±0.31	2.48±0.18*	3.04±0.35	3.14±0.29

Maximum length (second row), protrusion rate (third row) and force exerted by filopodia (fourth row) in control conditions (second column), in the presence of 10 μM EH (third column),of 20 μM EH (fourth column), of 25 μM CK (fifth column) and of 50 μM CK (sixth column). The student t-test has shown that data significantly differ with respect to control conditions

*P<0.05 and

**P<0.005. Data represent mean ± SEM.

**Fig 5:**
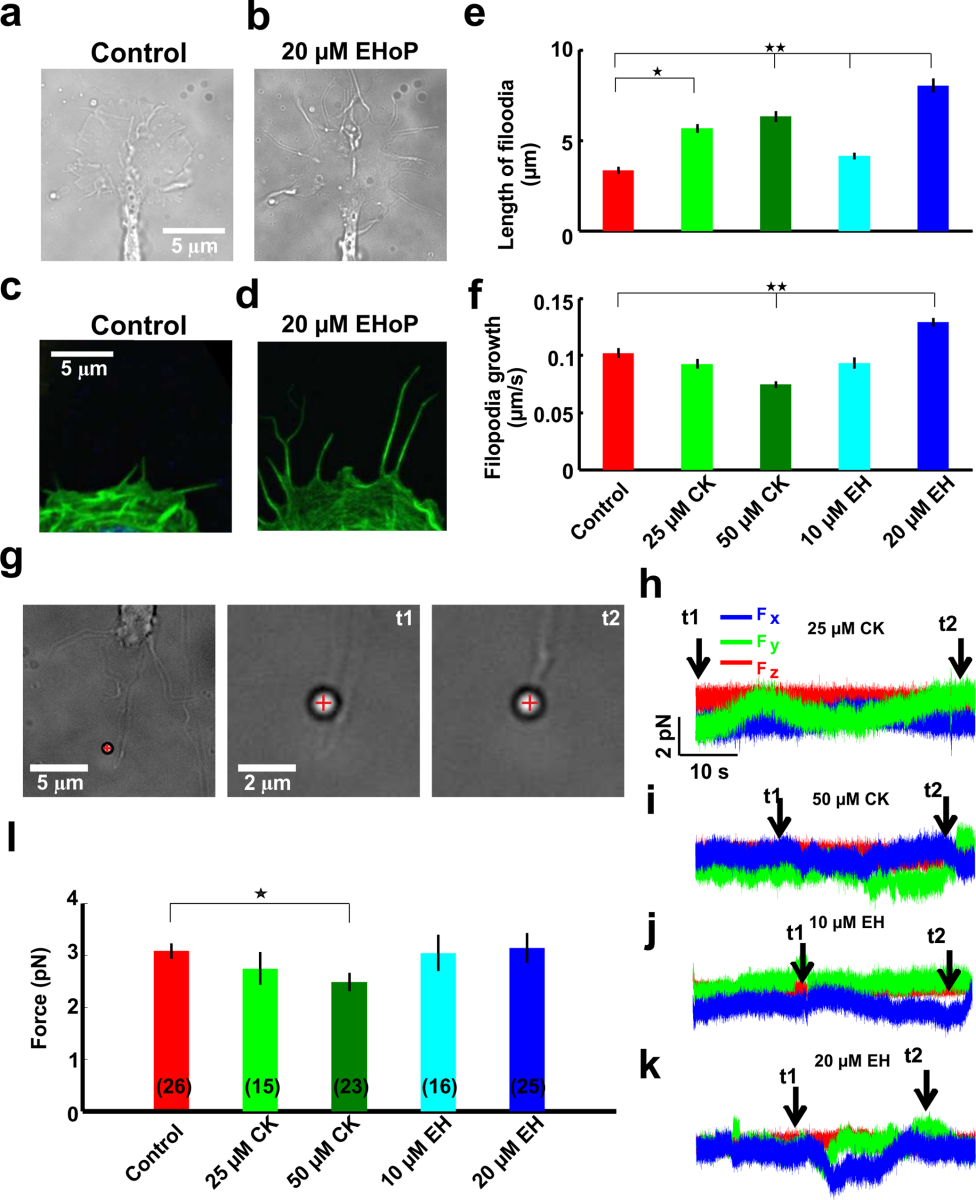
The effect of the CK and EH on the motility and force exerted by filopodia. **(a-b)** Phase contrast images of GC before and after treatment with 20 μM EH. Note the length of filopodia in each case. Scale bar 5 μm. **(c-d)** Staining of F-actin by phalloidin in GC before and after treatment with 20 μM EH. **(e)** Rate of filopodia protrusion in control conditions (red), with 25 μM CK (green), with 50 μM CK (dark green), with 10 μM EH (cyan) and with 20 μM EH (blue). **(f)** Maximum length of filopodia in control conditions (red), with 25 μM CK(green), with 50 μM CK(dark green), with 10 μM EH (cyan) and with 20 μM EH (blue). **(g)** Images of a bead trapped in front of a filopodium emerging from a GC of DRG neuron in the presence of 25 μM CK. At t_1_ the bead is in the optical trap and at t_2_ the filopodium pushes the bead. The cross indicates the centre of the optical trap. (**h)** The three components F_x_, F_y_ and F_z_ of the force exerted by the filopodium in the presence of 25 μM CK. **(i-k)** As in (h) but in the presence of 50 μM CK (i), in the presence of 10 μM EH (j) and in the presence of 20 μM EH (k) respectively. **(l)** Filopodia force in control conditions (red), in the presence of 25 μM CK (green), of 50 μM CK (dark green), of 10 μM EH (cyne) and of 20 μM EH (blue). The trap stiffness was k_x,y_ = 0.10 pN/nm, k_z_ = 0.08 pN/nm. By using the student t-test, the data differs with respect to the control conditions with a significance of *P<0.05 and **P<0.005. Data represent mean ± SEM. All the data were checked with chi-square test for Normal distribution before applying the student’s t test.

In DRG GC the length of the filopodia increased by 60 to 80% when Arp2/3 was inhibited by 25 and 50 μM CK respectively. When Rac1 was inhibited by 10 μM EH the length of the filopodia increased by 20%. Remarkably, the filopodia length increased more than the double when Rac1 was inhibited by 20 μM EH compared to control conditions (Fig 5A, 5B and 5E). The GCs were then fixed and stained with Alexa 488 phalloidin and imaged to observe the actin localization. The longer filopodia protruded from the GCs after the inhibition of Rac1 with 20 μM EH and showed an increase in the total F-actin compared to the controlled filopodia (Fig 5C and 5D).

The protrusion rate of filopodia did not change when Rac1 and Arp2/3 were suppressed by their respective inhibitors with a lower concentration. However, it increased by 30% when Rac1 was inhibited by 20 μM EH. In this case, the extension of the filopodia length could be the effect of this increase in the filopodia protrusion rate together with the decrease of the lamellipodia retrograde flow rate. Surprisingly, the filopodia protrusion rate decreased by 30% when Arp2/3 was inhibited by 50 μM CK (Fig 5F).

Inhibition of Rac1 and Arp2/3 significantly decreased the force exerted by lamellipodia; however, the force exerted by filopodia did not change when Rac1 was inhibited and, with a lower concentration of its inhibitor, Arp2/3 was suppressed, compared to control conditions. Very rarely filopodia emerged from lamellipodia exerted a force that is larger than 4 pN in control conditions. The forces exerted by filopodia were measured in the same neuron before and after the addition of inhibitors of Rac1 or Arp2/3. In each case collected data from 10 neurons showed that the filopodia force did not changed when Rac1 was inhibited by 10–20 μM EH and when the Arp2/3 was inhibited by 25 μM CK. Inhibition of Arp2/3 with 50 μM CK decreased the filopodia force by 20% when compared to control conditions (Fig 5L).

## Discussion

In this study we have characterized the role of Rac1 and Arp2/3 in the motility and force exerted by lamellipodia and filopodia of DRG GCs. Our results suggest that in neuronal growth cones, Rac1 acts as a switch that activates following the inhibition of Arp2/3. Moreover, Arp2/3 and Rac1 not only control the force exerted by lamellipodia but also the dynamics of filopodia.

### The effect of the inhibition of Rac1 and Arp2/3 on lamellipodia motility

**Fig 6:**
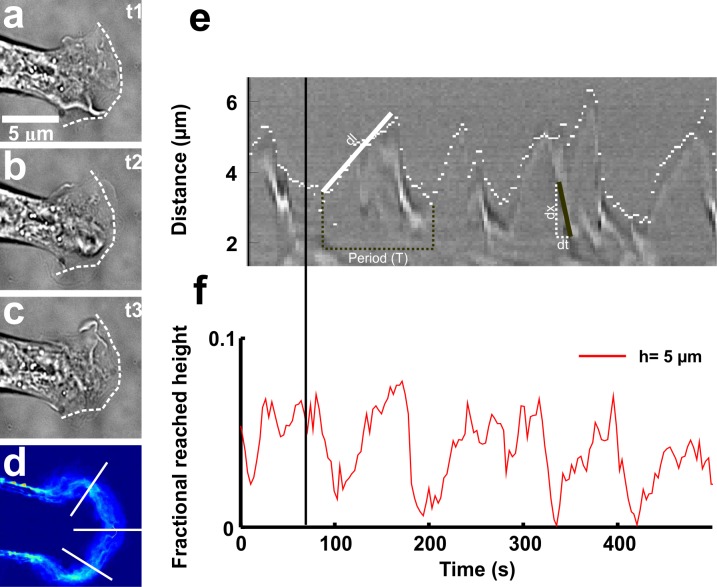
Characterization of lamellipodial protrusion/retraction cycles and of vertical motion. **(a-c)** From top to bottom: images of the lamellipodium undergoing cyclic waves of protrusion (t_2_) and retraction (t_1_ and t_3_) in control conditions; the white dotted line represents the leading edge of the lamellipodium_._ Scale bar, 5 μm. **(d)** The profile diagram of the positions of the lamellipodium edge during the time course. Increase in the color intensity shows increase in the frequency of the lamellipodia edge to be present at particular location. White lines used to plot the kymographs. **(e)** Kymograph showing the protrusion/retraction cycles of lamellipodia. White dots show the leading edge of lamellipodia. The characteristic values of period of protrusion/retraction cycles of lamellipodia motion (black dotted line), the retrograde flow rate (black line) and persistence length of lamellipodia (white line) i.e. (T), (dx/dt) and (dl) respectively were calculated along the label lines. **(f)** Fractional number of pixels in focus at 5μm height above the coverslip. The protrusion/retraction cycles of lamellipodia is also observed in terms of fractional reached height by lamellipodia. The black line shows the peak position of the fractional height where lamellipodia reach the maximum in axial direction at the end of the retraction.

We followed and quantified the protrusion/retraction cycles of DRG lamellipodia by measuring their period, persistence length and retrograde flow rate using kymographs (see Fig 6 in the Materials and Methods section).

Lamellipodia treated with a small amount of Rac1 and Arp2/3 inhibitors increased the period of their protrusion/retraction cycles (Fig 1C). When Rac1 was inhibited, the lamellipodia retrograde flow rate decreased, leading to a longer retraction time and overall cycle period. These effects were then returned to the control level after the washout of the Rac1 inhibitor, 20 μM EH indicating that the inhibitor was not toxic to the neuronal growth cone. When Arp2/3 was inhibited, the lamellipodia retrograde flow rate remained constant but the persistence length increased. The combination of these two effects increased the period of protrusion/retraction cycle (Fig 1C–1E).

The actin retrograde flow level decreased after the Arp2/3 complex was knocked down with siRNA in primary cultured hippocampal neurons and neuroblastoma cells [11] but increased when the Arp2/3 complex was inhibited by CK666 and CK869[10]. We found that partial inhibition of Arp2/3 with 50 μM CK548 (Fig 1B), did not affect the lamellipodia retrograde flow rate after the recovery of lamellipodium motility.

### Recovery of motility following partial inhibition of Arp2/3

When Arp2/3 was inhibited by 100 μM CK548 the growth cone dynamics was completely abolished presumably because of the loss of adhesion. However, when Arp2/3 was partially inhibited by 50 μM CK548, lamellipodia transiently shrank for 5–8 minutes but then recovered their usual motility. The Rho family of GTPase signaling proteins plays a pivotal role in regulating actin cytoskeleton [15] and could be involved in the observed recovery of lamellipodia motility. The best characterized small GTPases of the Rho family are Rac1, Cdc42 and RhoA which act as molecular switches, cycling between an active GTP-bound state and an inactive GDP-bound state [40]. To determine the possible role of Rho GTPase signaling pathways, in the transient retraction and recovery of lamellipodia when Arp2/3 was inhibited, we used selective inhibitors of Rac1, Cdc42 and RhoA.

Lamellipodia treated with the inhibitor of Rac1, EH showed an increase in their period of protrusion/retraction cycle and could move in the axial direction. The lamellipodia regained their motility after the washout of the EH, which show that the inhibitor effect on the neuronal growth cone was not due by its toxicity. When the same lamellipodia were later treated also with 50 μM CK548, lamellipodia showed the usual retraction but did not recover even after 10–20 minutes (Fig 2B). Moreover, when treated with both Rac1 and Arp2/3 inhibitors together, lamellipodia shrank as usual but again they did not recover after 10–20 minutes of exposure (Fig 2C). The higher concentration of EH not only inhibit Rac1 but also Rac3 and Cdc42 in the MDA-MB-435 metastatic cancer cells[35]. To rule out the possibility that the Rac3 and Cdc42 could be behind the recovery of transiently retracted lamellipodia in the Arp2/3 inhibited condition we tested the effect of the specific inhibitor of Cdc42, ZCL and of another specific inhibitor of Rac1, F56. Transiently retracted lamellipodia following partial Arp2/3 inhibition condition did recover also in the presence of the Cdc42 inhibitor, ZCL (Fig 2D). Transiently retracted lamellipodia following Arp2/3 inhibition did not recover when treated also with 100 μM F56.

The level of activated Rac1 following an exposure to 50 μM CK for 8 minutes significantly increased compared to what observed in control conditions, 25 μM CK(2 min) and 50 μM CK(2 min) (Fig 2I). These results indicate the specific role of Rac1 in the observed recovery of motility following partial inhibition of Arp2/3.

A possible mechanism could be mediated by the Integrin pathways. Jacquemet, G. *et al*. suggested that the engagement of integrin followed by filamin-A, IQGAP1 and RacGAP1 enrollment, deactivates Rac1[42]. Ilić, D. *et* al. and Saunders, R. M. *et al*. reported that Arp2/3 is recruited to nascent integrin adhesions through interaction with FAK and vinculin, where it reinforces the link between integrin and the cytoskeleton [43,44]. Furthermore, Beckham et al. reported that Arp2/3 inhibition impairs integrin, an extracellular membrane attachment resulting in either a translocation or treadmilling of mature adhesions [45]. Therefore, it is possible that inhibition of Arp2/3 could reduce the ligation and clustering of integrins and further suppress filamin-A, IQGAP1 and RacGAP1 recruitment, leading to an enhancement of Rac1 activity. Rac1 not only regulates actin polymerization but also increases the free actin-barbed ends and actin monomers. Therefore, the enhanced Rac1 activity could promote the formation and protrusion of lamellipodia, stimulating Arp2/3 by activating the WASP/WAVE family proteins [12,13].To study the role of other Rho GTPase pathways in the transient retraction and recovery of lamellipodia upon Arp2/3 inactivation, inhibitors of the respective pathways were used. Detailed quantification showed that the lamellipodia persistence length significantly increased after CT and GSK treatment but it remained constant after ZCL treatment, which is also consistent with what previously reported [46]. The increase in the lamellipodia persistence length upon CT and GSK treatment is probably due to the crosstalk between RhoA and Rac1 [47]. The lamellipodia retrograde flow rate significantly decreased after ZCL treatment; however, it remained constant when treated with CT and GSK. As previously shown, CDC42 promotes retrograde flow rate [48] thus the observed significant decrease in the lamellipodia retrograde flow rate is the direct result of CDC42 inhibition. Lamellipodia treated with CDC42 inhibitor increased the period of their protrusion/retraction cycles (Fig 2F). When CDC42 was inhibited, the lamellipodia retrograde flow rate decreased, leading to a longer retraction time and overall cycle period. When RhoA and ROCK were inhibited, the lamellipodia retrograde flow rate remained constant but the persistence length increased. The combination of these two effects increased the period of protrusion/retraction cycle (Fig 2F). These results show that inhibitors of CDC42, RhoA and ROCK were functioning appropriately. In addition to that, in all these cases lamellipodia showed recovery when treated with Cdc42, RhoA and ROCK inhibitors before treatment with Arp 2/3 inhibitor. Therefore, the involvement of these pathways in the recovery of lamellipodia motility can be discarded.

### Arp2/3 controls the formation and dynamics of filopodia

In the active states Rac1, Cdc42 and RhoA interact not only with their specific downstream targets but also cross talk [15]. Specifically, activation of Cdc42 triggers a localized activation of Rac1, initiating the filopodia formation [49]. In our experiments the presence of actin was confirmed in the filopodia before and after the inhibition of Rac1 by using immunocytochemistry (Fig 5). Inhibition of Rac1 remarkably increased the protrusion speed as well as the maximum length of the filopodia (Fig 5E and 5F). Since Rac1 inhibition reduces the activation of Arp2/3, it is possible that Rac1 inhibition decreases the formation and protrusion of lamellipodia, leaving filopodia behind. In addition, a decrease in the lamellipodia retrograde flow rate and a stable persistence length due to Rac1 inhibition (Fig 1D and 1E) are expected to cause an accumulation of actin at the peripheral region of the GC, from where the filopodia emerge. A higher concentration of actin at the base of the filopodia enables the growth of substantially longer filopodia[50,51].

Korobova et al. found that inhibition of Arp2/3 reduced the lamellipodia protrusion as well as filopodia formation and dynamics [11]. In our case we found that Arp 2/3 inhibition decreased the protrusion speed of filopodia but it increased their maximum length (Fig 5E and 5F). Inhibition of Arp2/3, increased the lamellipodia persistence length and the retrograde flow rate (Fig 1D and 1E), which will accumulate less actin at the periphery of the GC. This may possibly lead to a decrease in the protrusion speed of filopodia. Moreover, Arp2/3 is required for the formation of filopodia and inhibition of Arp2/3 could decrease the formation of new filopodia. The actin accumulated at the periphery of the growth cone upon Arp2/3 inhibition can be utilized by the remaining filopodia to form longer filopodia. We also found that when Arp2/3 was inhibited, the force exerted by filopodia decreased compared to control conditions. The above results indicate the direct involvement of the Arp2/3 in the formation and dynamics of filopodia. On the other hand, Rac1 inhibition increased the length of filopodia but it did not change the force they exerted. This indicates that, like Arp2/3, Rac1 may not directly take part in the formation and dynamics of filopodia (Fig 5E–5I).

In conclusion, we show here that Rac1 activates when Arp2/3 is inhibited possibly through the Integrin pathways acting as a feedback. Besides its role in lamellipodia formation, Arp2/3 is directly involved in the formation and dynamics of filopodia, while Rac1 is not involved in the activity of the force generation of filopodia.

## Materials and Methods

### Neuron preparation

Wistar rats at postnatal days 10–12 (P10-P12) were sacrificed by decapitation after anesthesia with CO_2_ in accordance with the Italian Animal Welfare Act. The Ethics Committee of the International School for Advanced Studies (SISSA-ISAS) has approved the protocol (Prot.n. 289-II/7). After dissection, Dorsal Root Ganglia (DRG) were incubated with trypsin (0.5 mg/ml; Sigma-Aldrich, Milan, Italy), collagenase (1mg/ml; Sigma-Aldrich) and DNase (0.1 mg/ml; Sigma-Aldrich) in 5 ml Neurobasal medium (Gibco, Invitrogen, Milan, Italy) in a shaking bath (37°C, 35–40 min). After mechanical dissociation, they were centrifuged at 300 rpm, resuspended in the culture medium and plated on poly-L-lysine-coated (0.5 μg/ml; Sigma-Aldrich) coverslips. Neurons were incubated for 24–48 h and nerve growth factor (50 ng/ml; Alomone Labs, Jerusalem, Israel) was added before performing the measurements.

### Quantification of lamellipodia and filopodia motility

Neurons were maintained at 37°C in the sample holder of the microscope stage capable of moving in X and Y directions with nanometer precision and imaged through 100 X oil immersed, 1.4 NA objective lens mounted on an inverted microscope (IX80, Olympus). Stacks of phase contrast images of neurons from DRG ganglia were obtained by Charge couple device (CCD) camera (Olympus Megaview) and by moving the objective lens vertically. Each stack contains images obtained in the focal plane of the objective, focused on the coverslip where neurons were cultured i.e. at height 0 and at 1, 2, 3, 4, 5 and 6 micron above the coverslip. Stacks of images were acquired with 0.1–1 Hz frequency to quantify the 3D motion of lamellipodia. Then, for a further analysis, the time lapse image sequence for each height was extracted by using Xcellence software (Olympus) to create videos of different height. Two algorithms were developed to quantify the dynamics of lamellipodia. Algorithm I was designed to quantify in a semi-automatic way the time course of protrusion/retraction cycles by using an improved version of the Kymograph [52,53]. Algorithm II was designed to quantify the vertical motion of lamellipodia during these cycles.

### Algorithm I

The images at height ‘0’ i.e. the cover slip where neurons were cultured- were focused and were used to analyze the protrusion/retraction cycles of lamellipodia (Fig 6). The lamellipodia edges were extracted from each image of the video by using the difference of Gaussian filter [54]. Lamellipodia edges were tracked and followed during the entire duration of the video (Fig 6A). A profile of the temporal movement of the lamellipodium edge was obtained. These profiles allowed to follow and quantify lamellipodia cycles of protrusion and retraction (Fig 6D). Then the regions of interest of each line were cut and lined up with the time course, to obtain separate kymographs corresponding to each line (Fig 6E).

The white dotted line in the kymograph shows the lamellipodia leading edge. The changes in the grey values show lamellipodia movements. Mainly the ascending white dotted parts of the dotted line show the protrusion of lamellipodia (white line showing single protrusion) while the descending white dotted parts of the line represent the retraction of lamellipodia. The time to complete one protrusion and retraction by the lamellipodia was considered as a period (T) of protrusion/retraction cycle of lamellipodia. The maximum protrusion length after which lamellipodia starts retracting (white line, dl; micrometers) was defined as the persistence length of lamellipodia. The dark appearances in the kymograph during each retraction of lamellipodia represent the retrogradely moving lamellipodia features (black line showing single lamellipodium retrograde flow). The slope of the lines drawn on these dark appearances was calculated to find out the lamellipodium retrograde flow rate (dx/dt; micrometer per second) [52,53] (Fig 6E). Each parameter, the lamellipodia period of the protrusion/retraction cycles, the persistence length and the retrograde flow rate, were calculated by extracting these features from many kymographs and averaged over for statistical significance.

### Algorithm II

Lamellipodia not only show periodic motion of protrusion and retraction (Fig 6C) but, during retraction, they also lift up and ruffle. To study the axial motion of GC lamellipodia, image sequences taken at different heights i.e. 0, 1, 2…6 were acquired and analysed. Algorithm II was based on the theory of defocusing, in which a pixel is assumed to be in focus at a specific height when its intensity equalises with the background intensity of the image of that height [55]. The background intensity of the image for each height was computed as the median of pixel intensities of the image for that height. The number of pixels in focus at a specific height was obtained and normalized by dividing it by the total number of pixels in focus at all the given heights. In this way, the fraction of pixels of the lamellipodium in focus at different heights, was extracted and plotted against time (Fig 6F). In this manner it was possible to study the maximal height reached by the lamellipodia edge during retraction in different conditions. Usually lamellipodia lift up high around the maximal retraction, so, in our experiments, their cyclic motility could be characterized both by the kymograph and by the fractional height that was reached (Figs 2 and 6).

In order to quantify the motility of filopodia, phase contrast time lapse image sequences acquired at height ‘0’ were analyzed. An Imagej (Image processing and analysis in Java) software was used to measure the maximum length of the filopodia and plug-in, ‘manual tracking’ was used to identify the protrusion rate of the filopodia.

### Force Measurements

The force exerted by lamellipodia and filopodia was calculated by measuring the displacement of the optically trapped bead and the known trap stiffness[32]. Unlike traction force microscopy or other similar measurement methods, initially, the bead was not in contact with the cell membrane [24,27,56,57] but was kept in the vicinity of the motile lamellipodia and filopodia. In this way the lamellipodia and filopodia can displace the bead in a spontaneous manner.

The Optical Tweezers (OT) set-up used for force measurements was as previously described[29,32]. The optical tweezers set-up was built as described in Ref. 31.

### Nanopositioner feedback

**Fig 7:**
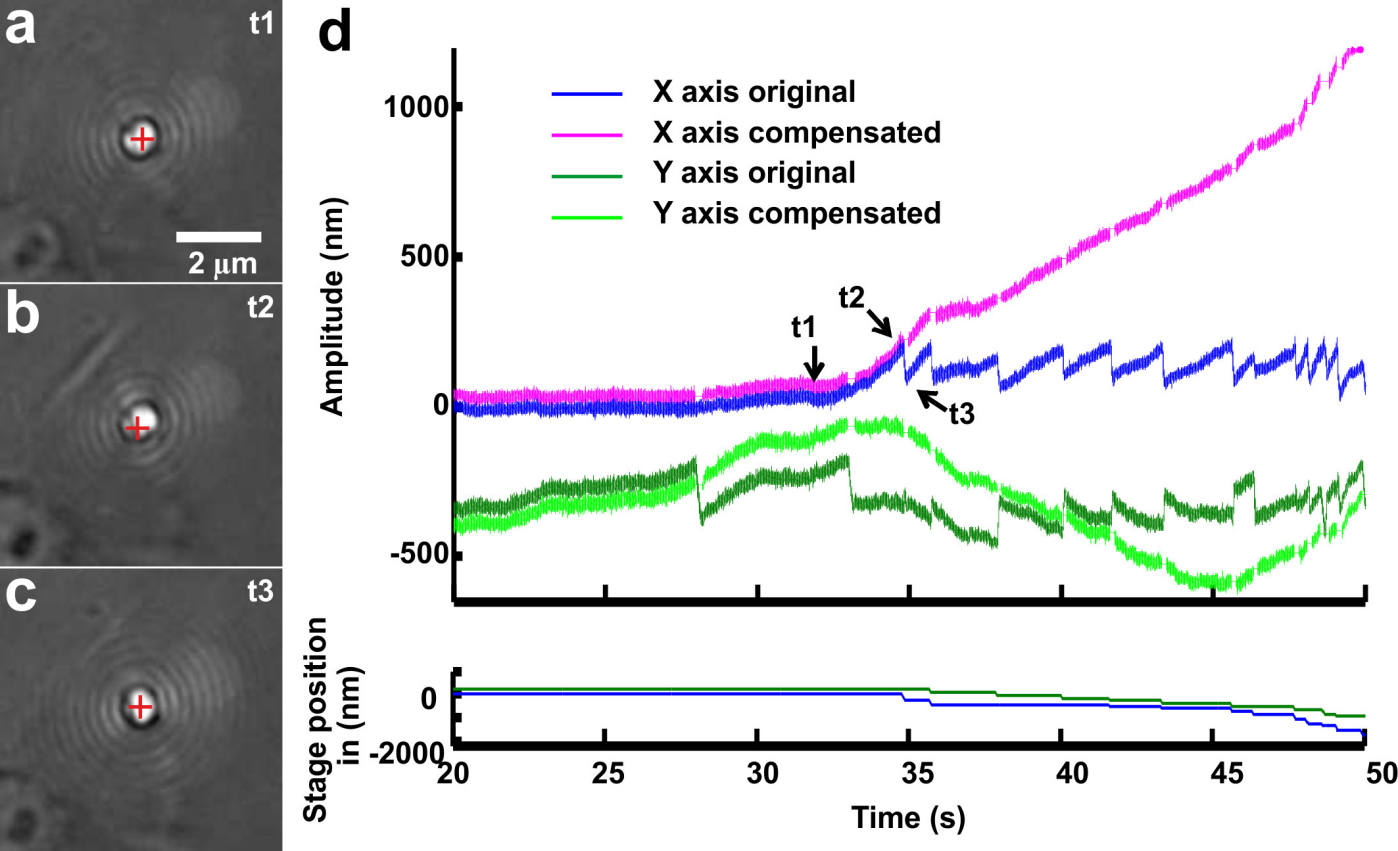
The feedback and nanopositioner system. (a-c) High-resolution images of a bead trapped in front of a lamellipodium emerging from the soma of a DRG neuron in control conditions and during a push. At t_1_ the bead is in the optical trap (a) Scale bar, 2μm.The lamellipodium grows, at t_2_, tries to push the bead out of the trap (b). At t_3_ the feedback mechanism of the Nano-drive redirects the bead back into the centre of the trap (c). The red cross indicates the center of the optical trap. (d) The X, Y components of the trace. The position of the bead (blue and green curve respectively, upper panel), compensated the X, Y position of the bead (magenta and light green curve, upper panel), corresponding to X, Y position of the Nanodrive (Blue and green respectively, lower panel).

In the OT setup, the detection of the position of the bead was based on the interference signal in the back focal plane, monitored with Quadrant Photo Detector (QPD) [58]. Often lamellipodia were able to push the bead out of the linear range–typically 200 nm—in which the QPD could provide a reliable measurement. To overcome this situation, a feedback mechanism, based on a nanopositioner stage-Nanodrive (Mad City Labs, USA) was used (Fig 7).

To summarize, from the detected x and y coordinates of the bead the displacement ‘r’ of the bead position from the centre of the trap was computed as sqrt (x^2+y^2). The nanodrive stage brings back the bead into the centre of the optical trap when r is larger than the threshold (which is usually set to be equal to 200 nm). By using the information of the displacement of the nanodrive stage (Fig 7D, lower panel) and the bead position determined by the QPD (X, Y axis original in Fig 7D) we recovered the x-y axis of the compensated displacement.

### Immunostaining

Cells were fixed in 4% paraformaldehyde containing 0.15% picric acid in phosphate-buffered saline (PBS), saturated with 0.1 M glycine, permeabilized with 0.1% Triton X-100, saturated with 0.5% BSA in PBS (all from Sigma-Aldrich, St.Louis, MO) and then incubated for 1h with primary antibodies. The secondary antibodies were goat anti-rabbit 594 Alexa (Invitrogen, Life Technologies, Gaithersburg, MD, USA) and anti-mouse IgG_2a_ biotynilated (Santa Cruz Biotechnology, Santa Cruz, CA) and the incubation time was 30 min. F-actin was marked with Alexa Fluor 488 phalloidin, whereas biotin was identified by Marina Blue-Streptavidin (Invitrogen, Life Technologies, Gaithersburg, MD, USA) and incubated for 30 min. All the incubations were performed at room temperature (20–22°C). Cells were examined using a Leica DMIRE2 confocal microscope (Leica Microsystems GmbH, Germany) equipped with DIC and fluorescence optics, diode laser 405nm, Ar/ArKr 488nm and He/Ne 543/594nm lasers. The fluorescence images (1024x1024 pixels) were collected with a 63X magnification and 1.3 NA oil-immersion objective. Leica LCS Lite and Image J by W. Rasband (developed at the U.S. National Institutes of Health and available at http://rsbweb.nih.gov/ij/) were used for image processing.

### Rac1 activity assay

The Rac1-GTP level (the activated form of Rac1) was determined in DRG neurons in control conditions, 25 μM CK (2 min), 50 μM CK (2 min) and 50 μM CK (8 min) using the G-LISA Rac 1 activation assay kit (Cytoskeleton, Inc., Denver, CO, catalog number BK128) according to the manufacturer’s instructions.

After experimental treatment, neurons were washed with ice-cold (4°C) PBS and then lysed in ice-cold lysis buffer. The lysate was clarified at 10000 x g at 4°C for 1 min, a 20 μl aliquot was taken for a protein assay, and the remaining lysate was separated into at least two aliquots, snap frozen in liquid nitrogen, and stored at −70°C until the start of the ELISA portion of the assay. Protein concentrations were determined using the Precision Red Advanced Protein Assay that came with the kit. Absorption of the ELISA wells was determined with a Multiskan™ GO Microplate Spectrophotometer (Thermoscientific, USA).
